# Towards Inclusive Fiscal Policy: A Disability-Responsive Taxation Framework for Equity and Economic Empowerment

**DOI:** 10.3390/ijerph23060736

**Published:** 2026-05-31

**Authors:** Michael Mncedisi Willie, Siyabonga Jikwana, Onke Ronaldy Mnyaka, Wezile Wilson Chitha, Khona Dyantyi

**Affiliations:** 1 Policy Research and Monitoring, Council for Medical Schemes, Pretoria 0157, Gauteng, South Africa; m.willie@medicalschemes.co.za; 2 Gauteng Department of Health, Johannesburg 2001, Gauteng, South Africa; siyabonga.jikwana@gauteng.gov.za; 3 School of Public Health, University of Pretoria, Pretoria 0084, Gauteng, South Africa; 4 Institute of Clinical Governance & Healthcare Administration, School of Public Health, Walter Sisulu University, Mthatha 5117, Eastern Cape, South Africa; wchitha@wsu.ac.za; 5 Department of Occupational Therapy, University of KwaZulu-Natal, Durban 4041, KwaZulu-Natal, South Africa; khona60@gmail.com

**Keywords:** disability-responsive taxation, fiscal inclusion, economic inequality, disability grants, administrative accessibility, South Africa

## Abstract

**Highlights:**

**Public health relevance—How does this work relate to a public health issue?**

- Disability affects income, healthcare access, and social protection, influencing health outcomes.
- Inequitable tax systems can worsen financial and health vulnerabilities for persons with disabilities.

**Public health significance—Why is this work of significance to public health?**

- Highlights how fiscal policies shape social determinants of health and equity.
- Supports inclusive policy design to reduce disability-related health disparities.

**Public health implications—What are the key implications or messages for practitioners, policy makers and/or researchers in public health?**

- Policymakers can design tax policies that improve access to resources and health outcomes.
- Researchers and practitioners can integrate fiscal strategies into broader health equity interventions.

**Abstract:**

Introduction: Disability in South Africa remains a key driver of socioeconomic inequality, affecting labour market participation, income security, and access to social protection. Conventional fiscal instruments, including medical tax credits and deductions, favour formally employed, higher-income taxpayers, leaving many persons with disabilities fiscally excluded. This study used a mixed-methods secondary analysis of peer-reviewed literature, policy documents, labour force data, disability grant records, and household cost estimates to develop a conceptual framework for disability-responsive fiscal inclusion. Results: Labour force data indicate that 10.2% of individuals outside the labour force are due to illness or disability, while discouraged jobseekers rose from 15.2% (2016) to 20.6% (2025). Households with severe disabilities face opportunity costs estimated at R2441 per month from lost earnings, caregiving, transport, and medical expenses. Disability grant patterns show male dominance in permanent disability grants for ages 18–45, with females surpassing males at 50–60. Temporary disability grants follow similar trends, with male predominance in the 18–35 age range and female predominance in the 40–60 age range. These findings reveal systematic gender- and age-related inequities in access to fiscal relief. Conclusions: Existing tax measures insufficiently address the financial burden of disability, disproportionately favouring urban, formally employed households. Implementing refundable tax credits, simplifying administrative processes, and adopting gender- and age-sensitive policies can enhance fiscal inclusion, reduce inequities, and strengthen economic participation for persons with disabilities in South Africa. This study proposes a framework to guide policymakers in implementing refundable disability tax credits, simplifying administrative processes, and targeting vulnerable groups, including older women, rural households, and low-income earners, to enhance fiscal inclusion, equity, and access to essential services.

## 1. Introduction

Disability significantly shapes socioeconomic inequality by influencing income security, labour market participation, and access to social protection systems. However, South Africa’s fiscal architecture is largely designed around assumptions of stable employment, predictable income streams, and standardised household expenditure patterns, which do not reflect the lived realities of persons with disabilities [1,2,3]. As a result, many individuals experience irregular or interrupted employment trajectories and incur additional disability-related costs, thereby reducing the redistributive effectiveness of conventional income tax and social protection mechanisms.

Those outside formal employment are particularly exposed to fiscal exclusion, a challenge further intensified by high levels of income inequality, persistent unemployment, and the expansion of the informal economy [4,5,6]. In addition, geographic disparities further shape fiscal accessibility and administrative reach, as urban residents often face higher living costs and complex bureaucratic systems, while rural households encounter infrastructural deficits and limited digital access to services [3].

Within a rights-based socioeconomic framework, disability is understood as arising from the interaction between impairments and environmental, attitudinal, and institutional barriers rather than being solely a health condition. In this context, evidence shows that people with disabilities in South Africa continue to face significant structural and institutional barriers to employability, while existing social protection measures remain fragmented and insufficient, leaving persistent gaps in both labour market inclusion and economic support [2,6].

South African evidence reinforces this position by demonstrating that disadvantage is produced through the combined effects of impairment, structural inequality, and systemic exclusion [1]. In this regard, “structural barriers” refer to system-level constraints such as inaccessible public services, labour market exclusion, discriminatory practices, and fragmented or inefficient social protection systems, including complex administrative procedures and delayed payments that restrict access to income support and employment opportunities [2].

These structural constraints are closely linked to administrative barriers and service access challenges, particularly where implementation inefficiencies and bureaucratic processes further hinder participation. Empirical evidence also shows that these interconnected barriers impose significant financial and economic burdens on households with persons with disabilities, including additional transport, healthcare, and care-related costs, which in turn reinforce cycles of inequality and dependency [3].

Persons with disabilities consistently experience lower labour market participation, reduced lifetime earnings, and higher poverty risk across both high- and middle-income countries [7,8,9]. In South Africa, structural barriers further restrict employment opportunities, reducing access to income-based tax relief [10,11]. Disability-related expenses, including healthcare, assistive technologies, transport, and personal assistance, create additional financial pressure [1,3,8]. Although medical tax credits and deductions partially address these costs, they disproportionately benefit higher-income, formally employed individuals, while lower-income households struggle due to liquidity constraints and administrative hurdles [3,4,5].

Administrative complexity further limits the effectiveness of tax systems for persons with disabilities. Documentation requirements, reporting procedures, and reliance on intermediaries create barriers, particularly for those with health limitations or limited digital literacy [1,12]. Disability outcomes are also influenced by gender and age [2,11,13]; younger men often experience higher disability prevalence from occupational hazards, while women encounter increasing prevalence later in life due to cumulative health burdens and longevity. Uniform fiscal assumptions can exacerbate inequities, leaving older women with interrupted labour histories and higher costs particularly disadvantaged [3,5,11]. Existing tax credits, largely focused on medical expenses, capture only part of these burdens and tend to favour urban and formally employed populations [12].

Despite extensive research linking disability to poverty and its additional costs, taxation remains an underexplored tool for inclusion. The literature has largely focused on social grants and expenditure-side interventions, often treating taxation as a technical fiscal mechanism rather than a rights-based instrument for equity and inclusion [3,6,9,14]. In South Africa, despite strong constitutional commitments to equality, limited integration between public finance, disability studies, and social policy has resulted in a fragmented understanding of fiscal tools and their role in disability inclusion [2,10].

Within this context, refundable tax credits are increasingly recognised as a more inclusive form of support, particularly for low-income households and persons with disabilities excluded from non-refundable tax relief, with evidence also suggesting that combining them with in-kind assistance can improve welfare and developmental outcomes among low-income families [15,16].

South African evidence on the feasibility and contextual application of refundable tax credits remains limited, despite international research indicating that they are more equitable and efficient than deductions or exclusions, as they decouple benefits from tax liability and strengthen redistribution [17].

Evidence on housing policy shows that well-designed tax credits can mobilize private investment and improve the delivery of affordable housing when supported by regulation, equity safeguards, and integration with existing programs to prevent exclusion and market distortions [18]. Broader studies further demonstrate that refundable tax credits reduce poverty, inequality, and household debt by increasing disposable income among low-income households [19]. In contrast, South Africa’s current medical and disability tax deductions offer limited relief for qualifying healthcare and disability-related costs [20], while administrative complexity, eligibility thresholds, and documentation requirements may constrain access, underscoring the need for research on more inclusive and scalable tax credit mechanisms.

This study therefore sought to develop a framework that integrates economic, equity, and rights-based perspectives to assess how tax systems can better support the inclusion of people with disabilities within broader social protection and labour market systems, while also further examining complementary social security mechanisms and contextual factors, such as access to in-kind benefits, labour market barriers, and institutional design, that shape the vulnerability and socioeconomic outcomes of this population.

## 2. Methods

This study employed a mixed-methods secondary analysis within an integrative policy analysis design to develop a conceptual framework on disability-related fiscal exclusion in South Africa. The approach is grounded in the premise that fiscal exclusion is a multidimensional phenomenon requiring the integration of multiple data sources and disciplinary perspectives [2,4,10], and this is reflected in the finding that structural disability-related exclusion remains a significant barrier to social and economic inclusion, with social assistance systems such as those administered by South African Social Security Agency (SASSA) playing a critical role in mitigating vulnerability and supporting basic livelihood security among affected populations [21,22].

Age- and gender-disaggregated disability grant data were obtained from SASSA [22], while labour market indicators, including participation rates, unemployment, median income, and poverty prevalence, were drawn from Statistics South Africa (StatsSA) [23,24]. Disability-related monthly costs, including healthcare, assistive devices, transportation, and personal assistance, were examined in conjunction, including healthcare, assistive devices, transportation, and personal assistance, with tax provisions, such as medical tax credits and deductions, to evaluate both eligibility criteria and administrative accessibility [1,3,9]. In line with this, Tjan [25] argues that progressive tax systems reduce income inequality through redistributive mechanisms; however, their effectiveness is shaped by policy design, enforcement capacity, administrative efficiency, and broader structural economic conditions.

The analysis integrated peer-reviewed literature on disability and social protection [1,5,6,20], fiscal policy frameworks [2,9,26], labour force survey data [23,24], administrative grant records [21], and published estimates of disability-related household costs [3,7] to construct a coherent, context-sensitive interpretation of fiscal processes affecting persons with disabilities. In addition, a systematic literature review was conducted to identify and synthesise scholarly and policy-relevant evidence on the intersection of disability, taxation, and social protection, using a structured and transparent search strategy across Scopus, Web of Science, PubMed, and Google Scholar, supplemented by targeted grey literature searches.

Search strategies combined controlled and free-text terms, including: “disability taxation”, “tax credits AND disability”, “medical tax deductions”, “disability grants South Africa”, “fiscal policy AND disability inclusion”, “social protection AND disability costs”, and “disability-related expenditure burden”. Boolean operators were applied to refine results and ensure conceptual precision.

Eligibility criteria were defined a priori. Studies were included if they (i) addressed disability in relation to fiscal systems, taxation, or social protection; (ii) contained empirical evidence, policy analysis, or theoretical development relevant to fiscal inclusion; and (iii) were published in English. Studies were excluded if they focused exclusively on biomedical or clinical outcomes without fiscal relevance, or if they lacked sufficient methodological or analytical grounding (e.g., opinion pieces without an evidential basis).

Study selection followed a three-stage screening process consisting of title screening, abstract review, and full-text assessment. Although not registered as a formal PRISMA review, the selection logic followed PRISMA-aligned principles of transparency, reproducibility, and systematic exclusion. The final corpus was subjected to thematic synthesis, focusing on patterns related to fiscal barriers, cost burdens of disability, and access constraints in tax and social protection systems.

To situate the empirical literature within institutional and regulatory realities, a structured policy analysis was conducted. This included an examination of South African fiscal and social protection instruments, such as medical tax credits, disability-related deductions, and disability grant frameworks administered by SASSA [22].

These were analysed alongside international policy frameworks on disability inclusion, social protection, and fiscal equity in order to benchmark South Africa’s approach against global normative standards [1,3,8]. The documentary analysis also enabled triangulation between formal policy intent and the practical accessibility of fiscal mechanisms.

Secondary quantitative data were incorporated to contextualise the analysis within real-world socioeconomic conditions affecting persons with disabilities. Labour market indicators, including employment rates, unemployment levels, income distribution, and poverty prevalence, were drawn from StatsSA [23,24]. These datasets provided macro-level insight into structural inequality and labour market exclusion.

Administrative data on disability grant receipt, disaggregated by age and gender, were obtained from the SASSA [22]. These data were used to examine patterns in the distribution of state support across demographic groups.

In addition, disability-related cost estimates were drawn from published empirical studies and national reports, capturing recurring monthly expenditures for healthcare, assistive devices, transportation, and personal assistance. These data were not subjected to econometric modelling; rather, they were used analytically to interpret the magnitude of disability-related financial burden in relation to fiscal relief mechanisms.

Data analysis followed an iterative and reflexive synthesis approach consistent with mixed-methods secondary policy analysis [2,4,10]. Evidence from peer-reviewed literature, policy documents, labor force statistics, administrative disability grant data, and published cost estimates was systematically compared, interrogated, and integrated through repeated cycles of conceptual mapping and cross-source triangulation [3,7,21,22,23]. This process enabled the identification of convergent and divergent patterns in the ways disability is represented, financed, and administratively managed within fiscal systems [1,5,6,17].

Through this synthesis, three analytically grounded dimensions emerged as central to understanding disability-related fiscal exclusion: substantive inclusion, referring to the extent to which fiscal instruments meaningfully reduce disability-related economic disadvantage; distributional equity, reflecting the fairness of fiscal benefits and burdens across population groups; and administrative feasibility, capturing the accessibility, clarity, and implementability of fiscal mechanisms in practice. These dimensions were not predetermined but were inductively derived from the integrated evidence base and subsequently refined through iterative validation across data sources [10,20,25].

Within the South African context, Willie [27] proposed a disability-responsive taxation (DRT) framework that explains fiscal exclusion through labour market inequality, disability-related costs, and limitations in tax and transfer systems. The study is conceptual and based on secondary evidence, requiring empirical validation across contexts to test its applicability. The framework integrates diverse evidence sources and adopts an interdisciplinary stance, bridging public finance concerns with disability rights perspectives while accounting for fiscal and administrative constraints.

## 3. Results

This section presents empirical findings that underpin the disability-responsive taxation framework. The results are organised into three analytical dimensions:

(i) income and labour market positioning,
(ii) disability-related costs and tax recognition, and
(iii) gender and age patterns.

The analysis focuses on structural patterns and how these conditions affect the reach and effectiveness of tax instruments for persons with disabilities.

### 3.1. Characteristics of the Population Outside the Labour Force

Labour market data confirm that persons with disabilities experience systematic disadvantage relative to persons without disabilities, constraining access to income-based tax relief [2,12]. Table 1 presents comparative indicators. Persons with disabilities participate in the figure illustrates notable shifts in the characteristics of individuals outside the labour force over the period 2016 to 2025. The proportion of students declined from 41.4% in 2016 to 35.8% in 2025, suggesting a relative reduction in education-related inactivity. Similarly, the share of homemakers decreased from 18.0% to 14.3%, indicating gradual changes in household and caregiving roles. The proportion of persons outside the labour force due to illness or disability remained relatively stable, decreasing slightly from 10.7% to 10.2%, which reflects persistent health-related barriers to labour market participation. In contrast, findings for those who are too old or too young to work show a modest increase, from 9.4% to 10.7%, pointing to growing demographic pressures. A significant change is observed among discouraged jobseekers, whose share rose from 15.2% in 2016 to 20.6% in 2025. This increase highlights worsening labour market conditions and rising levels of long-term discouragement. The “Other” category also expanded from 5.3% to 8.4%, suggesting greater heterogeneity in reasons for labour force exclusion.

### 3.2. Opportunity Costs for Households with Disabilities

Disability is associated with significant additional costs, many of which are only partially recognised within the South African tax system [1,11]. Households with severe disabilities have significantly lower total income compared to households without disabilities, despite receiving higher disability grants. The estimated opportunity cost, approximated as the difference in total household income, is about R2441 per month, reflecting income foregone due to reduced labour market participation and caregiving responsibilities. Households with broad disabilities receive slightly higher total income than non-disabled households, largely due to grant support, indicating that social protection partially offsets lost earnings [3].

Table 2 presents household and grant income by disability status in South Africa for 2015. Households with broad disability reported the highest total household income at R7121, compared to R6402 for households without disability. In contrast, households affected by severe disability had substantially lower total household income at R4956, indicating heightened economic vulnerability. Grant income was also considerably higher among households with disabilities, particularly severe disability households, which received R1341 compared to R818 for broad disability households and R552 for households without disabilities. These findings suggest that social grants play a critical compensatory role for households affected by disability, although they may not fully offset broader income disparities and disability-related economic burdens.

Households with persons with disabilities incur significant opportunity costs that reduce income and productivity. These include lost earnings, time spent on caregiving, transport and medical expenses, disrupted education, and lower work efficiency. Table 3 summarises the key categories and their economic impact, highlighting the multifaceted burden faced by affected households [3,7].

#### 3.2.1. Liquidity Constraints and Administrative Barriers

Tax relief mechanisms are often designed in a retrospective manner, requiring households to initially absorb out-of-pocket costs before any deductions or rebates can be claimed, which inherently disadvantages liquidity-constrained individuals and households with irregular or unstable incomes [3]. As a result, lower-income groups and those in precarious employment face structurally reduced and often delayed access to tax relief, limiting the real redistributive potential of these instruments [1]. This inequity is further compounded by administrative complexity and stringent documentation requirements, which consistently emerge as critical barriers to effective participation in tax and incentive systems.

The OECD emphasises that overly complex compliance procedures disproportionately exclude vulnerable and low-income groups, thereby weakening both uptake and the intended equity effects of tax policy [14]. In the South African context, similar patterns are evident in housing tax incentive programmes, where intricate application processes, extensive regulatory requirements, and burdensome documentation discourage participation, particularly among smaller developers, ultimately constraining policy effectiveness and reach [18]. Likewise, medical and disability-related tax deductions require taxpayers to maintain detailed supporting evidence, including receipts, medical certification, and proof of eligibility, which can be difficult to compile for individuals facing limited resources, lower tax literacy, or functional constraints [20].

#### 3.2.2. Gender and Age Disability Grants Distribution–2025

Analysis of disability grant data from January to August 2025 reveals pronounced gendered patterns across age groups, which have significant implications for fiscal inclusion and the equitable design of disability-responsive taxation [1,10]. gender between January and August 2025. The data show relatively stable administrative disbursements over time, with variation mainly observed across age cohorts and persistent differences between males and females throughout the period.

**Figure 1:**
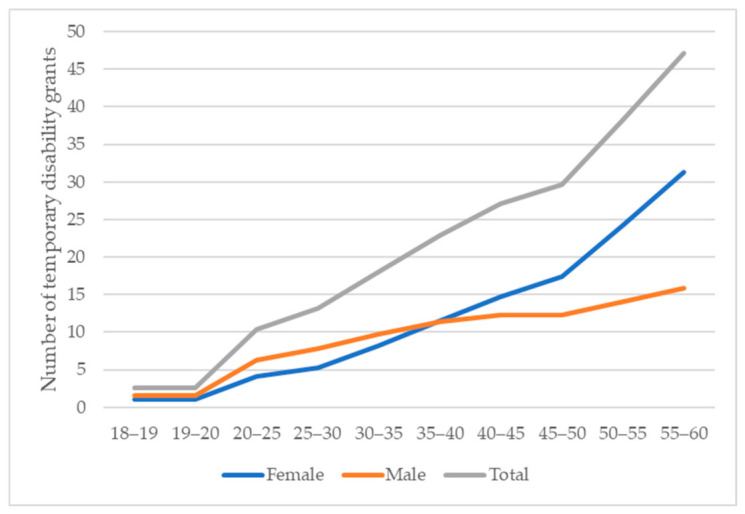
Number of temporary disability grants paid per month by age group and gender from January to August 2025, (Thousands) [22].

Figure 1 illustrates the monthly distribution of temporary disability grants disaggregated by age and gender [18].

**Figure 2:**
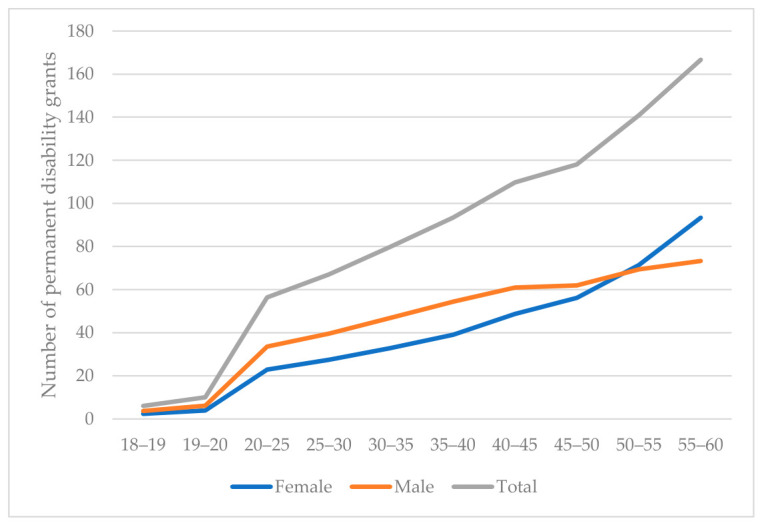
Number of permanent disability grants paid per month by age group and gender from January to August 2025, (Thousands) [22].

Figure 2 reflects a comparable pattern for permanent disability grants over the same timeframe, also stratified by age and gender.

Table 4 depicts a clear life-course pattern in gender differences across disability types, showing that gender dominance is not fixed but shifts across age groups. In both permanent and temporary disability, males consistently outnumber females in the younger cohorts (18–45 years for permanent disability and 18–35 years for temporary disability), which likely reflects earlier exposure to occupational risks, injury-prone environments, and labour market conditions associated with physically demanding work. However, this pattern changes in later life stages, where females begin to surpass males, particularly from ages 50–60 in permanent disability and 40–60 in temporary disability, suggesting a cumulative burden of health conditions and functional limitations among women as they age.

A distinct age–gender shift is observed in the distribution of permanent disability grants. While male beneficiaries predominate in younger working-age cohorts, this pattern reverses in later life, where female beneficiaries become more prevalent, particularly from ages 50–60. This transition suggests that gender disparities in disability are not static but evolve across the life course, reflecting changing exposure to risk, health trajectories, and survival patterns over time [9].

Temporary disability grants exhibit comparable trends but with a slightly earlier gender crossover. Males dominate younger age bands (18–35 years), consistent with episodic or short-term disability events typically linked to occupational hazards and accidents that disproportionately affect men. Female dominance begins to emerge from the 40–60 years age range, suggesting that women experience a growing burden of temporary disabilities during mid-to-late working age, potentially compounded by caregiving responsibilities, chronic health conditions, and workforce re-entry challenges [13].

These gendered and age-specific trends have direct fiscal implications. Since income-based tax relief and disability-related tax provisions are typically linked to regular employment and income, younger men with temporary or permanent disabilities are more likely to access these benefits. Conversely, older women with recurring or permanent disabilities are at heightened risk of fiscal exclusion, as they often experience interrupted or lower-paid labour market participation while facing elevated disability-related costs [2,10].

The distribution patterns of disability grants thus highlight the need for a differentiated, gender- and age-sensitive approach in designing disability-responsive taxation policies. Incorporating these insights ensures that fiscal measures do not inadvertently favour certain cohorts while leaving vulnerable groups, particularly older women, underserved.

#### 3.2.3. Proposed Expanded Disability-Responsive Taxation Framework

**Figure 3:**
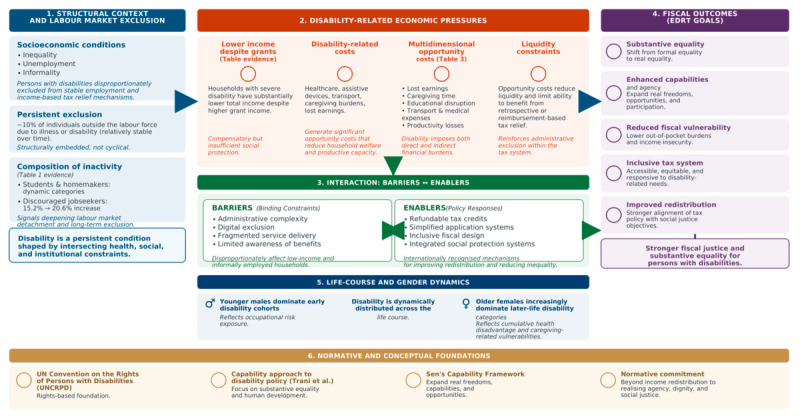
Expanded Disability-Responsive Taxation (EDRT) framework. Source: Adapted from Willie [27]; based on Sen [30], Trani et al. [29], and the UNCRPD [26].

The proposed Expanded Disability-Responsive Taxation (EDRT) framework (Figure 3) is grounded in the understanding that disability should not be reduced to an individual health condition but rather recognized as a structurally embedded determinant of labor market exclusion, income insecurity, and fiscal vulnerability. Building on Willie’s DRT framework, it extends the conceptualisation by incorporating empirical and theoretical evidence that highlights how these intersecting structural and socioeconomic constraints shape differential tax burdens and economic outcomes [1,2,3,27]. Within this framing, disability is not treated as an individual limitation alone but as an outcome of interacting social, economic, and institutional constraints that systematically shape unequal fiscal outcomes.

Empirical evidence supports this position. Winchester, King, and Rishworth [28] demonstrate that while social grants in Mpumalanga provide essential short-term financial relief, they remain insufficient to address deeper structural conditions of poverty, persistent economic precarity, and entrenched health inequities. Their findings further show that beneficiaries continue to experience multidimensional deprivation, reflecting the limits of income transfers in resolving broader structural exclusions within the labour market and social systems.

Building on these insights, the EDRT framework is grounded in rights-based and capability approaches. It draws explicitly on the United Nations Convention on the Rights of Persons with Disabilities (UN CRPD) [26], as well as Trani et al.’s application of the capability lens to disability policy and Sen’s capability framework [29,30]. Together, these foundations position disability-related fiscal policy within a broader normative commitment to substantive equality, focusing not only on income redistribution but also on expanding real freedoms, agency, and opportunities for persons with disabilities.

Within broader socioeconomic conditions characterised by inequality, unemployment, and informality, persons with disabilities remain disproportionately excluded from stable employment and income-based tax relief mechanisms [4,5]. This exclusion is reflected in the persistent share of individuals outside the labour force due to illness or disability, which remains relatively stable at approximately 10% over time, suggesting a structurally embedded rather than cyclical form of exclusion [23,24].

The composition of the population outside the labour force further substantiates the structural assumptions of the EDRT framework. Table 1 shows that while students and homemakers constitute dynamic categories of inactivity, the increase in discouraged jobseekers (15.2% to 20.6%) signals deepening labour market detachment and long-term exclusion [23,24]. This is consistent with evidence that disability and disadvantage intensify withdrawal from active labour market participation due to cumulative barriers [8,13]. The stability of disability-related inactivity confirms that disability is not merely a labour market status but a persistent condition shaped by intersecting health, social, and institutional constraints [10,21].

These structural conditions translate directly into the disability-related economic pressures component of the EDRT framework. Empirical evidence shows that households affected by severe disability experience substantially lower total income despite receiving higher grant income compared to both non-disabled and broadly disabled households [3]. This reflects the compensatory but insufficient nature of social protection in addressing structural income losses [6,7]. Disability-related costs, including healthcare, assistive devices, transport, caregiving burdens, and lost earnings, generate significant opportunity costs that reduce household welfare and productive capacity [3,7].

Table 3 further demonstrates that disability produces multidimensional opportunity costs, including lost earnings, caregiving time, educational disruption, transport and medical expenses, and productivity losses [22]. These findings align with evidence that disability imposes both direct and indirect financial burdens that are often underestimated in fiscal policy design [5,11]. Consequently, households face liquidity constraints that limit their ability to benefit from retrospective or reimbursement-based tax relief mechanisms, reinforcing administrative exclusion within the tax system [2,3,5,14].

Within the EDRT framework, the interaction between barriers and enablers becomes empirically evident. Administrative complexity, digital exclusion, fragmented service delivery, and limited awareness of benefits function as binding constraints that disproportionately affect low-income and informally employed households [14,18]. Conversely, enablers such as refundable tax credits, simplified application systems, inclusive fiscal design, and integrated social protection systems represent internationally recognised mechanisms for improving redistribution and reducing inequality [14].

The gender- and age-disaggregated disability grant data (Figure 1 and Figure 2; Table 4) further extend the framework by demonstrating that disability is dynamically distributed across the life course. Younger males dominate early disability cohorts, reflecting occupational risk exposure, while older females increasingly dominate later-life disability categories, reflecting cumulative health disadvantage and caregiving-related vulnerabilities [9,13]. These shifting patterns confirm the need for gender-sensitive and life-course-responsive fiscal policy design rather than static eligibility approaches.

## 4. Discussion

The findings of this study reinforce and extend existing evidence that persons with disabilities in South Africa continue to experience persistent and multidimensional economic disadvantage despite the presence of formal fiscal and social protection mechanisms [1,5,6,10]. This disadvantage is not only a function of individual impairment but is structurally produced through the interaction of labour market exclusion, administrative complexity, and disability-related costs embedded within fiscal systems [2,14,17]. In this regard, the study confirms that current tax instruments remain insufficiently responsive to the lived economic realities of disability and tend to favour formally employed and higher-income groups [4,14].

A key finding is the persistence of structural labour market exclusion, characterised by the continued marginalisation of individuals from meaningful participation in the labour market, alongside a growing prevalence of discouraged jobseekers, reflecting deepening barriers to labour market entry and sustained exclusion from economic opportunity [23,24].

This trend reflects not only cyclical unemployment dynamics but also long-term detachment from labour market participation, consistent with international evidence that disability interacts with structural inequality to produce sustained exclusion [8,13]. Within the EDRT framework, this finding corresponds to the “structural exclusion” dimension, which conceptualises disability as a persistent condition shaping income trajectories and tax capacity rather than a marginal fiscal category.

The analysis of household income and disability-related costs further demonstrates that existing fiscal measures only partially offset the economic burden of disability. Although households with disabilities may receive higher grant income, those affected by severe disability still report substantially lower total household income, highlighting the compensatory but insufficient nature of current transfers [22]. This reinforces evidence that disability generates both direct and indirect costs, including transport, healthcare, assistive devices, and productivity losses that are not fully captured within standard tax deduction systems [3,7]. Within the EDRT framework, these findings substantiate the “disability-related cost burden” dimension, which emphasises that fiscal exclusion arises not only from income insufficiency but also from unrecognised expenditure pressures.

Importantly, the results demonstrate that opportunity costs remain a critical but under-recognised dimension of fiscal inequality. Households affected by disability experience cumulative losses through reduced earnings, caregiving responsibilities, and disrupted educational and labour market pathways [3,8,9]. These opportunity costs highlight the limitations of conventional tax relief instruments, which are primarily designed around direct medical expenditures rather than broader socio-economic constraints. The EDRT framework improves on existing approaches by explicitly incorporating opportunity costs as a structural fiscal variable, thereby extending beyond narrow income-tax logic toward a more comprehensive equity-based assessment of disability impact.

The gender- and age-disaggregated disability grant patterns provide further empirical support for the framework’s life-course sensitivity. The findings show that younger males receive disability support in early adulthood, while females become more prominent recipients in later age groups [22]. This pattern is consistent with evidence that occupational risks disproportionately affect men in early working life, while women experience cumulative disadvantage and health-related vulnerabilities over time [10,13]. Within the EDRT framework, these findings validate the necessity of a dynamic rather than static approach to fiscal design. Unlike conventional tax systems that apply uniform eligibility rules, the framework incorporates demographic heterogeneity, recognising that disability-related fiscal needs evolve across the life course.

The interaction between administrative barriers and liquidity constraints further highlights why existing tax mechanisms remain insufficient. Tax relief is often retrospective, requiring upfront expenditure that excludes low-income and informally employed households from meaningful participation [4,14]. This aligns with broader evidence that administrative complexity functions as a hidden form of exclusion in tax systems, particularly for populations with limited digital access or reduced functional capacity [1,6]. The EDRT framework addresses this gap by introducing administrative feasibility as a core analytical pillar, shifting attention from formal eligibility to practical accessibility.

When synthesised, these findings demonstrate that disability-related fiscal exclusion is not adequately explained by conventional tax theory alone, which prioritises efficiency and redistribution without fully accounting for structural barriers, heterogeneity of need, and administrative constraints. The EDRT framework, therefore, offers a more comprehensive analytical lens by integrating structural exclusion, disability-related cost burdens, and administrative feasibility. This multidimensional structure allows for a more accurate representation of how fiscal systems interact with disability-related disadvantage in practice.

Compared to existing approaches, the EDRT framework provides a more suitable analytical and policy tool by moving beyond narrowly defined tax-relief instruments and incorporating broader socio-economic determinants of fiscal exclusion. In particular, it links taxation to lived inequality through opportunity costs, demographic variation, and system-level barriers. This allows the framework to bridge disability rights-based approaches and public finance approaches, thereby offering a more integrated and context-sensitive model for policy design [26,29,30].

The findings from this study reinforce that disability is not a marginal policy concern but a central determinant of fiscal inequality in South Africa. The EDRT framework, therefore, advances current scholarship by repositioning taxation as an instrument of substantive inclusion rather than a purely technical revenue mechanism. In doing so, it provides a stronger foundation for designing fiscal systems that respond to structural realities faced by persons with disabilities in contexts characterised by inequality, informality, and administrative fragmentation [4,14].

### 4.1. Study Limitations

This study has a number of limitations that should be considered when interpreting the findings. The analysis draws primarily on secondary data sources, including national surveys, disability grant administrative records, published empirical studies, and policy documents. While these sources provide a robust macro-level evidence base, they may not fully capture the diversity and complexity of lived experiences among persons with disabilities, particularly within informal settlements and rural contexts where administrative visibility and data coverage may be limited. In addition, disability-related cost estimates are derived from population-level averages, which necessarily mask substantial variation across households in health status, impairment severity, availability of support networks, and access to services such as healthcare, transport, and assistive technologies. Furthermore, administrative accessibility was inferred from documentary and statistical evidence rather than directly observed or measured, which limits the extent to which less visible barriers such as stigma, low levels of awareness, and digital exclusion can be empirically specified.

It is also important to acknowledge that the framework was developed without direct engagement with persons with disabilities or disability advocacy organisations. As a result, it does not fully operationalise the disability rights principle of “nothing about us without us,” which emphasises that research, policy design, and conceptual frameworks affecting persons with disabilities should be informed by their meaningful and active participation [25]. While both the DRT and EDRT frameworks are grounded in established disability theory, human rights-based approaches [27], and a synthesis of existing empirical literature, they have not yet been validated through participatory methodologies or grounded in lived-experience data.

Accordingly, the further refinement and empirical testing of the framework remain essential. Future research should prioritise participatory and co-produced approaches involving persons with disabilities, caregivers, and disability advocacy organisations to enhance the contextual relevance, ethical grounding, and practical applicability of the framework. Such work should include testing across diverse geographic, socioeconomic, and administrative settings, alongside iterative refinement informed by stakeholder engagement, to strengthen both conceptual validity and policy relevance.

Future research should also consider incorporating primary household-level data to complement the secondary evidence base, particularly to assess the actual utilisation and accessibility of disability-related tax benefits. Longitudinal analyses would further strengthen the understanding of how fiscal interventions influence poverty trajectories and economic participation over time. In addition, future studies should explore intersectional dynamics, particularly gender, age, disability type, and geography, in greater depth and extend comparative analysis to other low- and middle-income country contexts to further refine and strengthen the applicability of the EDRT framework for inclusive and equity-oriented fiscal policy design.

### 4.2. Recommendations

This study highlights the need to strengthen fiscal policy design so that it more accurately reflects the structural and economic realities experienced by persons with disabilities in South Africa. In particular, the findings support the refinement and expansion of refundable disability tax credits as a more inclusive instrument, especially for low-income and non-taxable households that are currently excluded from standard tax relief mechanisms. Such an approach is consistent with the broader evidence base on refundable tax credits as redistributive tools that extend fiscal support beyond formal employment structures and income thresholds.

In addition, the study underscores the importance of improving the accessibility and usability of existing fiscal instruments, particularly regarding administrative design. While existing policy frameworks already acknowledge administrative complexity as a barrier to access, the evidence reviewed in this study suggests that simplified application procedures, clearer eligibility pathways, and more accessible support channels could improve uptake. This is especially relevant in rural and underserved areas where limited infrastructure, lower digital connectivity, and constrained institutional reach may reduce effective access to fiscal support.

The results further indicate that disability-related fiscal measures should more explicitly reflect the heterogeneous nature of disability experiences across gender, age, and household context. In practice, this implies that policy design should avoid uniform assumptions and instead account for differentiated vulnerabilities, including older women with cumulative disadvantage, younger men affected by occupational injury, and households facing high levels of non-medical disability-related expenditure. This aligns with the empirical findings of the study, which demonstrate clear age–gender variation in disability grant distribution and labour market exclusion patterns.

## 5. Conclusions

Persons with disabilities in South Africa continue to face complex economic challenges, driven by labour market exclusion, elevated living costs, and barriers to accessing existing fiscal support. Current tax instruments provide only partial relief and disproportionately benefit higher-income, urban populations, leaving vulnerable groups underserved. The EDRT framework demonstrates that meaningful fiscal inclusion requires policies that integrate income realities, opportunity costs, demographic differences, and administrative feasibility. Effective, equity-focused fiscal measures can strengthen economic participation, reduce inequality, and support a socially just and inclusive tax system aligned with national priorities and international disability rights standards.

Policymakers may consider strengthening the use of secure, accessible digital platforms to improve the administration of tax-related services, with the aim of reducing administrative burdens for caregivers and households in rural and underserved areas. Such digital systems should be designed with robust security safeguards to protect against fraud, misuse, and other vulnerabilities within the service delivery environment. Ongoing policy discussions on income support mechanisms, including proposals such as a Basic Income Grant (BIG), should be aligned with broader fiscal reform efforts to ensure coherence, inclusivity, and integration with disability-responsive taxation measures.

## Figures and Tables

**Table 1:** Composition of the population outside the labour force.

	%	
	2016	2025
Students	41.4	35.8
Homemakers	18	14.3
Illness/Disability	10.7	10.2
Too old/Young to work	9.4	10.7
Discouraged jobseekers	15.2	20.6
Other	5.3	8.4

Source: StasSA (2016 vs. 2025) [23,24].

**Table 2:** Household and grants income by type, South Africa, 2015.

	ZAR (R)	
	Total Household Income	Grants Income
Without Disability	6402	552
Broad Disability	7121	818
Severe Disability	4956	1341

Source: UNICEF C Department of Social Development [3].

**Table 3:** Contributors to Opportunity Costs for Households with Disabilities.

Cost Category	Description/Impact
Lost Earnings	Reduced work due to caregiving or disability
Caregiving Time	Time spent on care instead of income-generating activities
Transport C Medical	Travel to services, medication, and assistive devices
Education Impact	Schooling delayed or disrupted to provide care
Productivity Loss	Stress and lower work efficiency

**Table 4:** Summary of Gender Dominance by Age Band.

Disability Type	Age Bands Where Males Surpass Females	Age Bands Where Females Surpass Males
Permanent disability	18–45 years	50–60 years
Temporary disability	18–35 years	40–60 years

## Data Availability

This study is based on secondary data sources, all of which have been appropriately cited within the manuscript. As such, no primary datasets were generated. The data supporting the findings of this study are available from the cited sources and, where applicable, may be obtained from the corresponding author upon reasonable request.

## Abbreviations

The following abbreviations are used in this manuscript:

BIG	Basic Income Grant
DRT	Disability-Responsive Taxation
DRTF	Disability-Responsive Taxation Framework
OECD	Organization for Economic Co-operation and Development
UN CRPD	United Nations Convention on the Rights of Persons with Disabilities
SASSA	South African Social Security Agency
